# AMPK Deficiency Increases DNA Methylation and Aggravates Colorectal Tumorigenesis in AOM/DSS Mice

**DOI:** 10.3390/genes15070835

**Published:** 2024-06-25

**Authors:** Qi Sun, Qiyu Tian, Alejandro Bravo Iniguez, Xiaofei Sun, Hui Zhang, Jeanene Deavila, Min Du, Mei-Jun Zhu

**Affiliations:** 1 School of Food Science, Washington State University, Pullman, WA 99164, USA; qi.sun6@wsu.edu (Q.S.); qiyu.tian@wsu.edu (Q.T.); a.bravoiniguez@wsu.edu (A.B.I.); xiaofei.sun@wsu.edu (X.S.); 2 Pharmaceutical Sciences, Washington State University, Spokane, WA 99202, USA; hzhang@wsu.edu; 3 Department of Animal Science, Washington State University, Pullman, WA 99164, USA; deavilaj@wsu.edu (J.D.); min.du@wsu.edu (M.D.)

**Keywords:** AMPK, colorectal cancer, epigenetic modification, α-ketoglutarate

## Abstract

The incidence of colorectal cancer (CRC) is closely linked to metabolic diseases. Accumulating evidence suggests the regulatory role of AMP-activated protein kinase (AMPK) in cancer metabolic reprogramming. In this study, wild-type and AMPK knockout mice were subjected to azoxymethane-induced and dextran sulfate sodium (AOM/DSS)-promoted colitis-associated CRC induction. A stable AMPK-deficient Caco-2 cell line was also established for the mechanistic studies. The data showed that AMPK deficiency accelerated CRC development, characterized by increased tumor number, tumor size, and hyperplasia in AOM/DSS-treated mice. The aggravated colorectal tumorigenesis resulting from AMPK ablation was associated with reduced α-ketoglutarate production and ten-eleven translocation hydroxylase 2 (TET2) transcription, correlated with the reduced mismatch repair protein mutL homolog 1 (MLH1) protein. Furthermore, in AMPK-deficient Caco-2 cells, the mRNA expression of mismatch repair and tumor suppressor genes, intracellular α-ketoglutarate, and the protein level of TET2 were also downregulated. AMPK deficiency also increased hypermethylation in the CpG islands of *Mlh1* in both colonic tissues and Caco-2 cells. In conclusion, AMPK deficiency leads to reduced α-ketoglutarate concentration and elevates the suppressive epigenetic modifications of tumor suppressor genes in gut epithelial cells, thereby increasing the risk of colorectal tumorigenesis. Given the modifiable nature of AMPK activity, it holds promise as a prospective molecular target for the prevention and treatment of CRC.

## 1. Background

Colorectal cancer (CRC) is the second leading cause of cancer death [1]. Epidemiological studies reported that metabolic diseases render patients susceptible to CRC and its related mortality [2], indicating a close relationship between metabolic disorders and CRC incidence. Colonic tumorigenesis is the cumulative consequence of genetic and epigenetic alterations in the colonic epithelium. Abnormalities in epigenetic changes, such as DNA methylation and histone methylation, occur more frequently than genetic mutations in this context [3]. Over half of human genes possess promoters with CpG islands, and their expression is regulated by DNA methylation [4]. Aberrant methylation in the promoters of tumor suppressors or metastasis inhibitor genes leads to transcriptional silencing [4]. Several markers associated with CRC, such as MutL homolog 1 (*Mlh1*, a mismatch repair (MMR) gene), a disintegrin and metalloproteinase with thrombospondin motif 1 (*Adamts1*, an anti-angiogenic gene), myelin and lymphocyte (*Mal*, a T-cell differentiation gene), and O-6-methylguanine-DNA methyltransferase (*Mgmt*), exhibit hypermethylation in their promoter regions [4,5,6,7]. The ten-eleven translocation hydroxylases (TETs) play a critical role in DNA demethylation by catalyzing the conversion of 5-methylcytosine (5mC) to 5-hydroxymethyl-cytosine (5hmC). In various cancer types, such as breast cancer, melanoma, and gastric cancer, the expression of TETs is suppressed, which correlates with the hypermethylation of related anti-oncogenic genes [8,9,10].

Unlike normal colonic cells, CRC cells exhibit a metabolic shift from oxidative phosphorylation towards aerobic glycolysis, known as the Warburg effect [11]. This altered metabolic phenotype involves the downregulation of the tricarboxylic acid cycle and its intermediates, such as α-ketoglutarate, which reprograms the metabolic microenvironment to promote tumor initiation and development [12]. Emerging evidence has shed light on the potential role of metabolic enzymes and their metabolites in mediating carcinogenesis through epigenetic mechanisms [13,14,15]. Specifically, α-ketoglutarate is an obligate substrate for TET-mediated DNA demethylation, establishing a link between cellular metabolism and epigenetic modifications.

Natural polyphenolic compounds, such as quercetin [16], magnolol [17], and berberine [18], are known for their anti-CRC effects with the concomitant activation of AMP-activated protein kinase (AMPK). AMPK, a critical metabolic mediator, is a promising therapeutic target for enhancing intestinal barrier function and differentiation [19]. Conversely, epithelial-specific AMPK knockout (AMPK KO), as observed in AMPK Vil1Cre mice, increases cell proliferation [20]. However, the direct relationship between AMPK and colorectal tumorigenesis has not been examined. In this study, we generated AMPK transgenic mice with a specific KO of AMPK in intestinal epithelial cells and further examined the role of AMPK in metabolic alterations, epigenetic modifications, and colorectal tumorigenesis using an azoxymethane (AOM)/dextran sulfate sodium (DSS) induced-CRC mouse model, as well as in human colonic epithelial Caco-2 cells with AMPK deficiency.

## 2. Methods

### 2.1. Mouse Strains

The animal studies were conducted in compliance with the guidelines and approved protocol of the Institutional Animal Care and Use Committee (IACUC) at Washington State University. Wild-type C57BL/6J mice were purchased from Jackson Laboratory (Bar Harbor, ME, USA). Mice carrying the AMPKα1-floxed gene (Prkaa1^tm1.1Sjm^/J, Stock#: 014141, Jackson Laboratory) were cross-bred with Vil1Cre mice (B6.Cg-Tg(Vil1-cre/ERT2)23Syr/J, Stock#: 020282, Jackson Laboratory). The resulting Vil1Cre offspring included 6 homozygous AMPKα1 wt/wt (wild-type, WT), 7 heterozygous AMPKα1 flox/wt (*Prkaa1*^+/−^), and 9 homozygous AMPKα1 flox/flox (*Prkaa1*^−/−^). At 6 weeks of age, all mice were intraperitoneally injected with tamoxifen (75 mg/kg of body weight) in sunflower oil (Sigma, St. Louis, MO, USA) at weeks 0, 2, 4, and 8 of the study to induce CRC development as described below. The dose of tamoxifen was selected based on our preliminary trials and existing literature [21] to maintain a sufficient concentration in mice without reaching a lethal dose.

### 2.2. AOM/DSS-Induced CRC

CRC was induced as previously described with modifications [22]. AMPKα1-Vil1Cre cross-bred mice received an intraperitoneal injection of AOM (MRI Global Chemical, Kansas, MI, USA) at a dose of 10 mg/kg body weight three days after the first tamoxifen injection. One week after the AOM injection, mice were subjected to three cycles of DSS (MP biomedicals, CA, USA) induction. Each cycle lasted four weeks with a one-week administration of 1% (*w*/*v*) DSS in drinking water, followed by three weeks of regular drinking water. The disease activity index (DAI) scores were evaluated based on established criteria [20,23]. The mice were euthanized at 22 weeks of age to assess the impact of the AMPK KO on CRC incidence.

### 2.3. Tumor Analysis and Colon Sample Collection

At the time of necropsy, the mice were anesthetized, followed by cervical dislocation. The colon was carefully removed for the mice with CRC induction, flushed with phosphate-buffered saline, and opened longitudinally. The colon tissues were placed on a dissecting board (ThermoFisher Scientific, Waltham, MA, USA), and adenomatous polyp numbers were counted. Photographs of the colon samples were captured using a cell phone camera positioned perpendicular to the midline. These images were subsequently analyzed for colon length and polyp diameters using ImageJ software (Version 1.53) [24]. The adenomatous polyp load per mouse was calculated as the sum of the diameters of all adenomatous polyps [22].

To assess tissue pathological changes, 0.5 cm distal colons near the anus were fixed in 4% formaldehyde and embedded in paraffin. The remaining colonic tissues were rapidly frozen in liquid nitrogen and stored at −80 °C for subsequent biochemical analyses.

### 2.4. Histopathological Analysis

The embedded distal colons were sectioned at a thickness of 5 μm and stained with hematoxylin and eosin stain, as previously reported [25,26]. The histopathological scores of the AOM/DSS-treated mice were evaluated following the described methods [27]. Inflammation, epithelial defect, crypt atrophy, hyperplasia, and hyperplasia area were considered histopathological parameters, and their severity was scored on a scale of 0 to 4, as previously specified [28].

### 2.5. Cell Culture

The human colonic epithelial Caco-2 cell line was obtained from the American Type Culture Collection (Manassas, VA, USA) and cultured in Dulbecco’s Modified Eagle’s medium (Sigma) supplemented with 10% fetal bovine serum (GE, Fairfield, CT, USA) and 100 units/mL penicillin-streptomycin (Life Technologies, Carlsbad, CA, USA). The cells were cultured at 37 °C with 5% CO_2_ in a humidified incubator. The transfected Caco-2 cells were selected, as previously described [20]. Briefly, Caco-2 cells were transfected with the green fluorescent protein (GFP) or the AMPKα K47R kinase-dead mutant (AMPK KD) plasmid (Addgene, Cambridge, MA, USA) [29]. The plasmids were introduced into Caco-2 cells using the Neon Transfection System (Invitrogen, Carlsbad, CA, USA) per the manufacturer’s instructions. Twelve hours after transfection, the medium was replaced, and 1000 μg/ mL G418 (Amresco, Solon, OH, USA) was added to the cells for 7 days to select and enrich the cells with successful transfection. The medium was changed daily during this selection period.

### 2.6. Immunoblotting Analysis

The frozen colonic tissues were powdered in liquid nitrogen. The protein extracts from the powdered colonic tissues or Caco-2 cells (cultured for 5 days) were separated using SDS-PAGE and transferred to a nitrocellulose membrane for immunoblotting [20]. The band density on the membrane was normalized to β-tubulin. Antibodies against phosphorylated acetyl-CoA carboxylase (p-ACC), phosphorylated AMPK (p-AMPK), AMPKα1, isocitrate dehydrogenase 1 (IDH1), MGMT, caudal type homeobox 2 (CDX2), proliferating cell nuclear antigen (PCNA), MLH1, mutS homolog 2 (MSH2), TET2, and MAL were purchased from Cell Signaling Technology (Danvers, MA, USA). The antibody against p53 was purchased from Santa Cruz Biotechnology (Dallas, TX, USA). The β-tubulin antibody was obtained from the Developmental Studies Hybridoma Bank, University of Iowa (Iowa City, IA, USA).

### 2.7. Immunohistochemical Staining

The immunohistochemical staining was performed as described previously with modifications [30,31]. Deparaffinized and rehydrated sections of mouse colon tissues were subjected to antigen retrieval via incubation in 10 mM citrate acid buffer (pH 6.0) for 10 min. To reduce non-specific binding, the section was blocked with 5% horse serum (Vector Laboratories, Burlingame, CA, USA) in phosphate-buffered saline solution with 0.05% Tween 20 (pH 7.4) for 1 h. The section was then incubated overnight at 4 °C with the PCNA antibody (Santa Cruz Biotechnology, Dallas, TX, USA) for immunohistochemical staining. Following primary antibody incubation, tissue sections were incubated with biotinylated secondary antibodies (1:200, Vector Laboratories) at room temperature for 30 min. PCNA-positive cells were visualized using Vectastain ABC and DAB peroxidase (HRP) substrate kits (Vector Laboratories), followed by hematoxylin counterstaining. The images of the stained sections were captured using a Lecia DM2000 LED light microscope (200×, Leica Microsystems, Wetzlar, Germany). The intensities of the PCNA-positive cells were quantified using ImageJ software (NIH). Briefly, the colon area was cropped, followed by color deconvolution (H-DAB). The intensity of the PCNA-positive cells was calculated by dividing the intensity of the brown color layer (representing specific DAB staining) by the density of the total colon section area.

### 2.8. Quantitative Reverse-Transcriptase (RT)-PCR

The total RNA was extracted from the colonic tissues using TRIzol (Sigma) following the manufacturer’s instructions. The extract RNA was then used for cDNA synthesis using the iScript cDNA Synthesis Kit (Bio-Rad, Hercules, CA, USA) according to the kit’s protocol. RT-PCRs were performed using the SYBR Green super mixture (Bio-Rad) on the CFX384 RT-PCR detection system (Bio-Rad). The primers, listed in Supplementary Table S1, were designed to span two exons to avoid the amplification of the genomic DNA. Lastly, 18s rRNA was used as an internal control. The relative changes in gene expression were determined using the 2^−ΔΔCt^ method [32].

### 2.9. α-Ketoglutarate Assays

The total α-ketoglutarate content in the powdered colonic tissues and intracellular α-ketoglutarate levels in the Caco-2 cells were tested using gas chromatography-mass spectrometry (GC-MS). The Caco-2 cells were cultured in 6-well plates for 2 days, collected, and homogenized in 1.2 mL of a solvent mixture (methanol: H_2_O = 2:1). For the colon tissue sample preparation, 15 μg of ground frozen colonic tissues were homogenized in 300 μL of the solvent mixture (methanol: H_2_O = 2:1). Ribitol was used as an internal standard at a concentration of 1.25 μg/mL (Sigma, St. Louis, MO, USA). The extracted metabolites were derivatized and analyzed using a GC-MS system as per our established method [19]. The GC-MS analysis was performed with helium as the carrier gas, a front inlet purge flow of 3 mL/min, and a gas flow rate of 1 mL/min through the column. The initial temperature was 50 °C and kept for 2 min, followed by temperature increases at a rate of 5 °C/min to 100 °C, which was held for 10 min, then increased at 10 °C/min to 200 °C, held for 10 min, and finally increased at 20 °C/min to 300 °C. The relative abundances of the target compounds were calculated by determining the area ratios of the target peaks to the ribitol peaks. The α-ketoglutarate standard used in the analysis was purchased from ThermoFisher Scientific (Waltham, MA, USA). The relative contents were calculated by comparing the abundance to those of the control group.

### 2.10. Methylated DNA Immunoprecipitation

Methylated DNA immunoprecipitation (IP) was performed as previously described with modifications [33,34]. Six homozygous WT and six homozygous AMPK KO mice (randomly picked from nine KO mice to facilitate the analysis) were used. The genomic DNA (10 µg) isolated from Caco-2 cells or colonic tissues was diluted in 300 µL of Tris-EDTA buffer and sonicated into 300–1000 bp fragments, denatured at 100 °C, then immediately cooled on an ice bath. The DNA fragment sizes were validated using agarose gel electrophoresis. The sonicated DNA solution was then incubated with a 1/10 volume of 10 × IP buffer and the respective antibody. The antibodies used included 5hmC antibody (Cell Signaling Technology), 5mC antibody (Zymo Research, Irvine, CA, USA), and normal rabbit IgG (Cell Signaling Technology) serving as a negative control. The DNA–antibody complex was incubated overnight at 4 °C and subsequently pulled down using pre-blocked Pierce™ magnetic protein G beads (Thermo Scientific, Waltham, MA, USA). The captured beads were washed three times with 1 × IP buffer and then resuspended in 250 µL of digestion buffer with proteinase K. Following treatment with proteinase K, the DNA was purified and concentrated, and subjected to RT-PCR analysis using the primers listed in Supplementary Table S1. The relative enrichment of 5hmC or 5mC was determined using 2^−ΔΔCt^, with ΔCt calculated as the change in the Ct value relative to the input DNA. 

### 2.11. Statistical Analyses

The data are presented as the mean ± standard error of the mean (SEM). Statistical significance was assessed using Student’s *t*-test with a two-tailed distribution and one-way ANOVA with Fisher’s least significant difference post hoc. A *p*-value ≤ 0.05 was considered statistically significant.

## 3. Results

### 3.1. AMPK Deficiency Aggravates Colorectal Tumorigenesis

**Figure 1:**
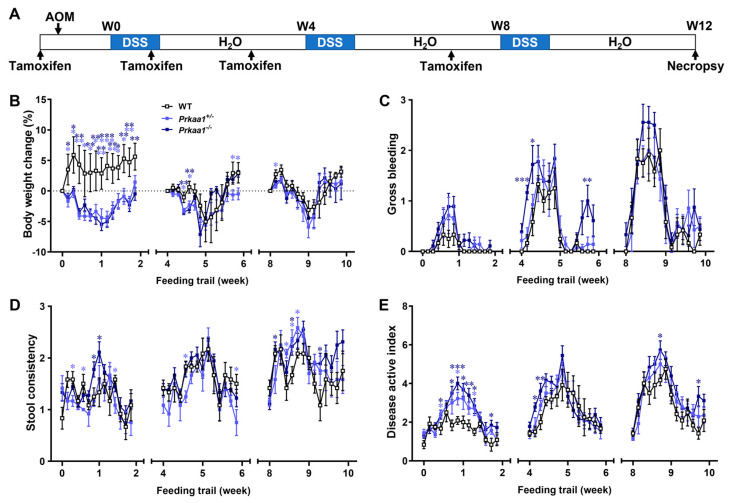
AMP-activated protein kinase (AMPK) ablation aggravates disease activity in an AOM/DSS-induced colorectal cancer mouse model. (**A**) The time frame: wild-type (WT) and AMPK intestinal epithelial cell-specific knockout heterozygous (*Prkaa1*^+/−^) and homozygous (*Prkaa1*^−/−^) mice at the age of 7 weeks were injected with azoxymethane (AOM, 10 mg/kg body weight) at the first day of experimental week 0 (W0). One-week post-AOM injection, the mice underwent 3 cycles of dextran sulfate sodium (DSS) induction. Each cycle involved providing 1.0% (*w*/*v*) DSS in drinking water for 7 days, followed by a 3-week recovery period. (**B**) Body weight change. (**C**) Fecal gross bleeding. (**D**) Stool consistency. (**E**) Disease activity index. Mean ± SEM, *n* = 6–9. *: *p* < 0.05; **: *p* < 0.01; ***: *p* < 0.001. A one-way ANOVA with Fisher’s least significant difference post hoc was used for the data analyses.

Villin-specific AMPK KO mice showed higher disease index scores, characterized by increased average weight loss, gross bleeding, and altered stool consistency, especially during the initial DSS induction and recovery cycle (Figure 1B–E).

**Figure 2:**
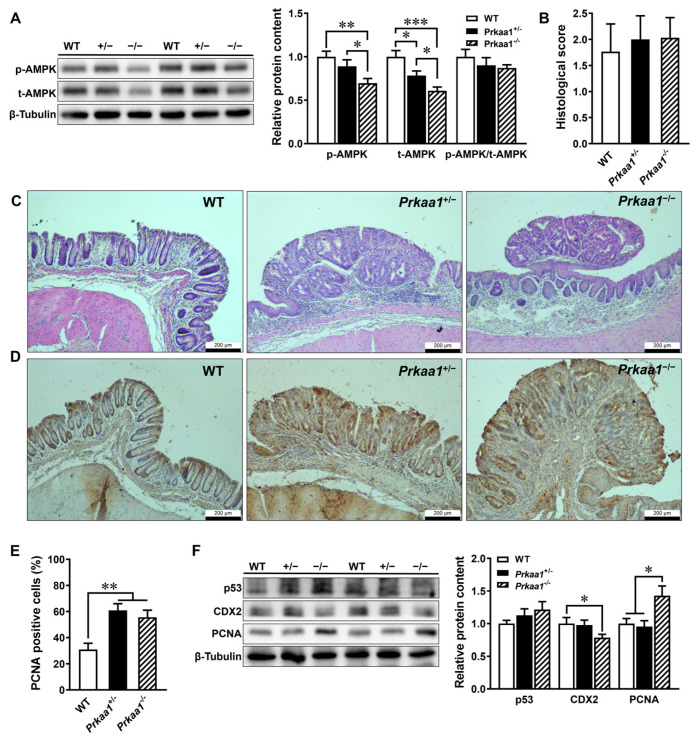
AMP-activated protein kinase (AMPK) deficiency accelerates colorectal tumorigenesis. Wild-type (WT) and AMPK intestinal epithelial cell-specific knockout (*Prkaa1*^+/−^) and homozygous (*Prkaa1*^−/−^) mice were induced to develop colon tumors with azoxymethane and dextran sulfate sodium and euthanized at 22 weeks of age. (**A**) Relative protein contents of phosphorylated AMPK (p-AMPK) and total AMPK (t-AMPK) in colonic tissues. (**B**) Histopathological scores of colonic tissues. (**C**) Representative hematoxylin and eosin staining of distal colonic tissues. The scale bars are 200 μm. (**D**) Representative images of immunohistochemistry staining of proliferating cell nuclear antigen (PCNA). The scale bars are 200 μm. (**E**) The percentage of the PCNA-positive area relative to the total epithelial tissue area was quantified using Image J. (**F**) Relative protein contents of p53, caudal type homeobox 2 (CDX2), and PCNA. Mean ± SEM, *n* = 6–9. *: *p* < 0.05; **: *p* < 0.01; ***: *p* < 0.001. A one-way ANOVA with Fisher’s least significant difference post hoc was used for the data analysis.

In this study, tumorigenesis was primarily observed in the distal colon and extended to the mid-colon in the majority of heterozygous (four out of seven) and homozygous (seven out of nine) AMPK KO mice, whereas only one WT mouse showed evidence of colon tumors (Figure S1A–D). Additionally, there was a trend of decreased colon length in the AMPK KO mice (Figure S1E). Histologically, AMPK inactivation (Figure 2A) triggered the formation of polyps in the distal colon (Figure 2C). There were no significant differences in the pathological scores among the different genotypes (Figure 2B,C). As expected, the heterozygous and homozygous AMPK KO mice had augmented proliferative cells (Figure 2D,E). The AMPK KO mice also had a lower expression of differentiation marker CDX2 and increased expression of proliferative marker PCNA, confirming the key role of AMPK in preventing tumorigenesis (Figure 2F).

### 3.2. AMPK Ablation-Induced CRC Is Associated with Decreased α-Ketoglutarate Production in Colonic Tissues

**Figure 3:**
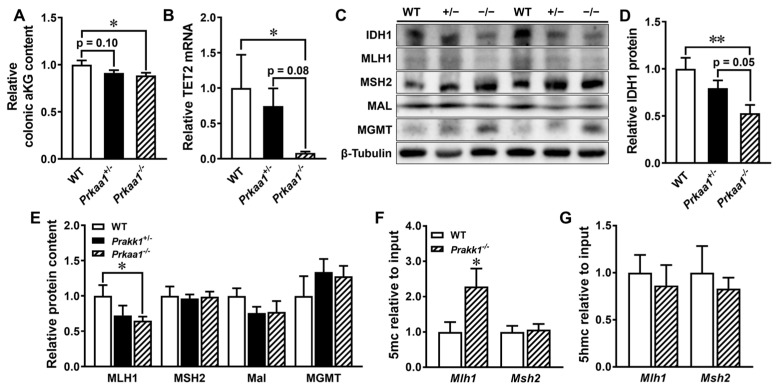
Loss of AMP-activated protein kinase (AMPK) inhibits α-ketoglutarate content, TET2, and tumor suppressors in the colonic tissues of azoxymethane (AOM)/dextran sulfate sodium (DSS)-induced mice. Wild-type (WT) and AMPK intestinal epithelial cell-specific knockout heterozygous (*Prkaa1*^+/−^) and homozygous (*Prkaa1*^−/−^) mice had induced colon tumors with AOM/DSS and were euthanized at 22 weeks of age. (**A**) Relative colonic α-ketoglutarate (aKG) content. (**B**) The mRNA level of ten-eleven translocation hydroxylase 2 (*Tet2*). (**C**) Representative immunoblots. (**D**) Relative protein contents of isocitrate dehydrogenase 1 (IDH1). (**E**) Relative protein contents of mutL homolog 1 (MLH1), mutS homolog 2 (MSH2), myelin and lymphocyte (MAL), and O-6-methylguanine-DNA methyltransferase (MGMT). (**F**) 5-methylcytosine (5mC) and (**G**) 5-hydroxymethyl-cytosine (5hmC) modifications in the CpG islands of *Mlh1* and *Msh2*. Mean ± SEM, *n* = 6–9, *: *p* < 0.05; **: *p* < 0.01. The data in A-E were analyzed using a one-way ANOVA with Fisher’s least significant difference post hoc and the data in F-G were analyzed using Student’s *t*-test with a two-tailed distribution.

Metabolic reprogramming is closely associated with tumorigenesis [35]. Mutations in IDH1, a critical metabolic enzyme catalyzing α-ketoglutarate formation, have been identified in patients with primary CRC [36]. Reduced intracellular α-ketoglutarate levels have been linked to AMPK deficiency [33]. Accordingly, IDH1 was downregulated in the colons of AMPK KO mice (Figure 3C,D), along with a decrease in its metabolic product, α-ketoglutarate (Figure 3A). The mRNA expression of TET2, which is involved in α-ketoglutarate-dependent DNA demethylation and can be phosphorylated by AMPK [37], was decreased in the colons of AMPK homozygous KO mice (Figure 3B). These changes were accompanied by alterations in the DNA MMR protein MLH1 (Figure 3C,E). The CpG regions of the MLH1 gene in the colons of AMPK KO mice displayed a higher methylation rate compared to WT mice (Figure 3F,G), which may be attributed to the deficiency of α-ketoglutarate/TET-dependent DNA demethylation pathways.

### 3.3. AMPK Regulates the DNA Methylation of Tumor Suppressive Genes in Caco-2 Cells

**Figure 4:**
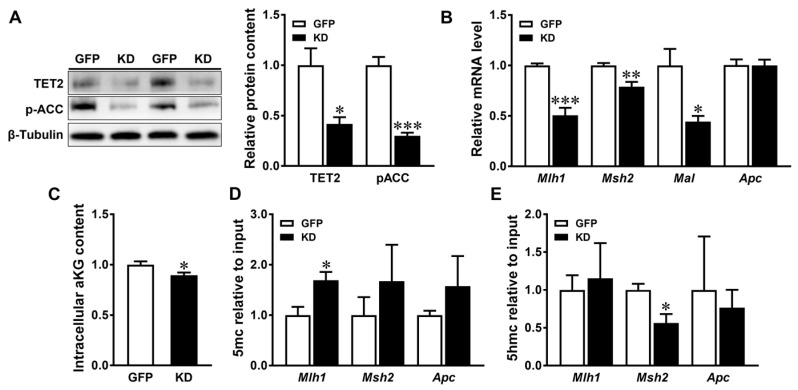
AMP-activated protein kinase (AMPK) prevents promoter hypermethylation in Caco-2 cells. GFP: cells transfected with the green fluorescent protein plasmid; KD: cells transfected with the AMPKα K47R kinase dead plasmid. (**A**) Relative protein contents of ten-eleven translocation hydroxylase 2 (TET2) and phosphorylated acetyl-CoA carboxylase (p-ACC). (**B**) mRNA expression of MutL homolog 1 (*Mlh1*), mutS homolog 2 (*Msh2*), myelin and lymphocyte (*Mal*), and adenomatous polyposis coli (*Apc*). (**C**) Relative intracellular α-ketoglutarate (aKG) content. (**D**) 5-methylcytosine (5mC) and (**E**) 5-hydroxymethyl-cytosine (5hmC) modifications in the promoters of *Mlh1*, *Msh2*, and *Apc*. Mean ± SEM, *n* = 3–4, *: *p* < 0.05; **: *p* < 0.01; ***: *p* < 0.001. The data were analyzed using Student’s *t*-test with a two-tailed distribution.

The deficiency of AMPK resulted in decreased AMPK kinase activity, demonstrated by the reduced levels of phosphorylated ACC, an AMPK substrate (Figure 4A). The ablation of AMPK also decreased the mRNA expression of key genes involved in DNA MMR, including *Mlh1*, *Msh2*, and *Mal* (Figure 4B). Furthermore, AMPK deficiency downregulated TET2 protein expression (Figure 4A), correlating with reduced intracellular α-ketoglutarate content (Figure 4C). Additionally, AMPK kinase deletion increased the 5mC abundance in the CpG region of *Mlh1* (Figure 4D) while decreasing the 5hmC abundance in the CpG regions of *Msh2* (Figure 4E). Collectively, these findings indicate that AMPK dysfunction induces epigenetic modifications, contributing to the acceleration of tumorigenesis.

## 4. Discussion

### 4.1. AMPK Deficiency Promotes Colorectal Carcinogenesis

CRC is one of the most prevalent and lethal forms of cancer [1]. Mounting evidence suggests that cancer development is closely associated with metabolic reprogramming. A meta-analysis encompassing over 2 million diabetic patients across 15 studies revealed that diabetic patients are susceptible to the incidence of CRC [38]. Obesity, a prominent risk factor for metabolic disorders, increased tumor growth in xenograft mice implanted with colon adenocarcinoma cancer cells [39]. Furthermore, obesity-induced alterations in systemic metabolism can modulate the tumor microenvironment, leading to the compromised infiltration and function of CD8+ T-cells [40].

Intestinal epithelial cells undergo continuous renewal with a turnover rate of every 4–5 days, a process requiring energy for sustained cell proliferation and differentiation [41]. AMPK, a critical mediator of cellular energy homeostasis, coordinates cellular metabolism to maintain a balance between energy demand and supply. As a metabolic checkpoint, AMPK determines whether the energy is adequate to proceed with cell division [42]. However, the dysregulation of AMPK causes metabolic disorders that can facilitate carcinogenesis [43]. AMPK inactivation has been associated with tumorigenesis in melanoma [44] and thyroid cancer [45], while its activation holds promise as a therapeutic target for preventing tumor development and progression in breast and hepatic cancers [46,47]. Metformin, a well-known anti-diabetic drug, is a pharmacological activator of AMPK. Epidemiological evidence suggests that metformin use in diabetic patients with CRC is associated with a reduced risk of cancer-related mortality [38]. Metformin suppressed the formation of aberrant crypt foci during early-stage CRC after mice were intraperitoneally injected with AOM six times [48] and reduced polyp size in the small intestine of Apc^min/+^ mice [49]. Furthermore, metformin attenuated DSS-induced acute colitis and ameliorated AOM/DSS-induced tumorigenesis in interleukin-10-deficiency mice [50]. Likewise, the chronic administration of metformin in mice slows the induction of pancreatic [51], breast [46], and lung cancers [52]. AMPK ablation decreases α-ketoglutarate production and TET activity, thereby impairing brown adipogenesis and thermogenesis [33]. The inactivation of TETs and subsequent loss of 5hmc contribute to DNA hypermethylation and tumorigenesis in melanoma [9]. In combination, these studies highlight the pivotal role of AMPK in bridging metabolism to epigenetic modifications and tumorigenesis.

During intestinal development, the delicate balance between epithelial proliferation and differentiation from intestinal stem cells is tightly regulated. The disturbance of this delicate balance can contribute to the progression of CRC. In the absence of AOM/DSS induction, we observed that AMPK ablation augmented proliferative cells in crypts, providing a possible potential explanation for the initiation of CRC. In vitro studies have demonstrated that AMPK activators, such as metformin and 5-aminoimidazole-4-carboxamide-1-β-D-ribofuranoside, can arrest the cell cycle and inhibit the proliferation of breast [46], hepatic [47], and prostate cancer cells [53]. Following AOM/DSS induction, AMPK deficiency aggravated colorectal tumorigenesis, as evidenced by the increased adenocarcinoma number, size, and hyperplasia in AMPK KO mice subjected to AOM/DSS treatment. Similarly, AMPK deficiency in the intestinal epithelium exaggerated DSS-induced colitis and histopathological scores [20]. These findings underscore the significant role of AMPK in regulating intestinal homeostasis and its impact on the development and progression of CRC.

### 4.2. AMPK KO-Induced α-Ketoglutarate Deficiency Induces Epigenetic Alterations Associated with CRC

α-Ketoglutarate, a prominent metabolite in the tricarboxylic acid cycle, plays a crucial role in connecting metabolism to epigenetic modifications [54]. Unlike normal epithelial cells, cancer cells predominantly rely on glycolysis rather than oxidative phosphorylation for energy production, a phenomenon known as the Warburg effect [55]. AMPK serves as a master metabolic regulator in rapidly proliferating cells such as cancer cells by activating the tricarboxylic acid cycle and inhibiting fatty acid synthesis [56,57]. In phosphatase and tensin homolog (PTEN)-deficient mice, AMPK inactivation was accompanied by a reduction in gene expression in the citric acid cycle, contributing to hyperplasia and hyperproliferation in the thyroid [45].

Aberrant DNA hypermethylation contributes to cancer cell growth and survival, while the DNA demethylation of specific genes leads to cell death and apoptosis [58]. TET enzymes play a crucial role in DNA demethylation, utilizing α-ketoglutarate as a co-factor [59]. TET proteins are known as 5mC hydroxylases, catalyzing iterative oxidation that results in the production of 5hmC, 5-formyl cytosine, and 5-carboxylcytosine [60]. These modified cytosine derivatives undergo DNA demethylation through the base excision repair pathway [61]. AMPK also directly phosphorylates TET2, enhancing its tumor-suppressive activity and modulating tumor suppression [37]. In this study, AMPK deletion resulted in a decreased level of IDH1, its catalytic product α-ketoglutarate, and the mRNA and protein levels of TET2, indicating the roles of AMPK in linking cellular metabolism and epigenetic modifications. Similarly, AMPK ablation reduced IDH2 activity and α-ketoglutarate levels in brown adipose tissue, thus decreasing TET activity and impeding DNA demethylation [33].

Aberrant methylation in the promoters of metastasis inhibitor genes leads to transcriptional silencing [4]. Methylation in the promoter regions of *Adamts1* and *Mal* serves as a biomarker for the early detection of CRC during stepwise tumorigenesis [5]. Similar to tumor suppressors, the DNA MMR genes *Mlh1* and *Msh2* play a critical role in DNA maintenance, and germline mutations in these genes are associated with hereditary nonpolyposis CRC/Lynch syndrome [62]. In line with these findings, we observed decreased MLH1 in the colons of AMPK KO mice and AMPK KD colonic epithelial cells. Our data provide evidence that AMPK deficiency exacerbates tumorigenesis and downregulated MLH1 in AMPK KO mice subjected to AOM/DSS induction and in AMPK KD cells. These changes are associated with hypermethylation in the CpG region of *Mlh1*. Furthermore, AMPK KD also downregulated the mRNA expression of *Msh2* and *Mal*. Importantly, the loss of AMPK kinase activity also downregulated the level of 5hmc in the CpG island of MSH2, suggesting that AMPK ablation inhibits DNA demethylation, possibly by suppressing TET2 activity. The reduction in MMR proteins or tumor suppressors due to AMPK ablation further underscores the protective effect of AMPK in tumorigenesis.

In summary, AMPK ablation induces hyperproliferation and accelerates the development of CRC, likely mediated through epigenetic modifications, which is associated with reduced IDH content, decreased α-ketoglutarate production, and impaired TET2 activity. These findings deepen our understanding of the link between AMPK, an intracellular energy sensor, and CRC. Given that AMPK is commonly inhibited in various pathophysiological conditions such as obesity and diabetes, which are known risk factors for CRC, targeting AMPK could be a promising strategy for mitigating intestinal tumorigenesis. Furthermore, the availability of drugs that activate AMPK, such as metformin, adds translational potential of our findings.

## Data Availability

The data presented in this study are available upon request.

## Supplementary Materials

The following supporting information can be downloaded at: https://www.mdpi.com/article/10.3390/genes15070835/s1, Figure S1: AMP-activated protein kinase (AMPK) knockout accelerates adenomatous polyps’ development in an AOM/DSS-induced CRC mouse model; Table S1: Primer sequences used for RT-qPCR analyses; Reference [63] is cited in the Supplementary Materials.
