# Supplementary material for: AMPK Deficiency Increases DNA Methylation and Aggravates Colorectal Tumorigenesis in AOM/DSS Mice
Source: Genes (Basel). 2024 Jun 25;15(7):835. doi: 10.3390/genes15070835 (PMC11276171; doi:10.3390/genes15070835)
Supplement: Supplementary file 1 [file genes-15-00835-s001.zip › genes-3051937-supplementary.pdf]

Supporting Information for Online Publication

Supporting Information Table S1. Primer sequences used for RT-qPCR analyses

Gene name	Accession No.	Product size (bp)	Sequence (5'→3')	Source
m*Tet2*	NM_001346736.1	71	F: ACAAATGACAGCACAACCGC<br>R: GAAGGTGCCTCTGGAGTGTT	This study
m*18s*	NR_003278.1	122	F: AAGACGGACCAGAGCGAAAG<br>R: ATCGCCAGTCGGCATCGTTT	[1]
h*Mlh1*	NM_000249.4	124	F: TGGAAATTGATGAGGAAGGGAAC<br>R: TCTTCGTCCCAATTCACCTC	This study
h*Msh2*	NM_000251.3	76	F: ACAGAATATGAAGAAGCCCAGG<br>R: GCATTGGTTCTACATAGCCTGA	This study
h*Mal*	NM_002371.4	105	F: CTGCCGTGGTGTTCTCCTA<br>R: CAGCTCAAGTTCTACTGCGG	This study
h*Apc*	NM_001407446.1	103	F: ACAAGGCTACGCTATGCTCT<br>R: CTCAAAACACTCGCAATAACCTG	This study
h*18s*	NR_003278.3	102	F: TTGTACACACCGCCCGTCGC<br>R: CTTCTCAGCGCTCCGCCAGG	[2]
CpG m*Mlh1*	NC_000075.7	120	F: GCTCCCTCCGTACCAGTTT<br>R: GTAGCAGGAGTTATTCGGCG	This study
CpG m*Msh2*	NC_000083.7	108	F: CTGCATGGTGATTGGGCGAG<br>R: CCACTAGAGCACAGAGCGAC	This study
CpG h*Mlh1*	NC_000003.12	105	F: TGTCCAATCAATAGCTGCCG<br>R: AAGTGCCTTCAGCCAATCAC	This study
CpG h*Msh2*	NC_000002.12	131	F: GTTGGTGCTTGTGCGGATT<br>R: CGACGTAAACACTCCGTGAT	This study
CpG h*Apc*	NC_000005.10	141	F: GGTCCTCCATTCTCACGCA<br>R: ACTAAAATGGGAAGGGCGGA	This study

References

1. Tian QY, Xu ZX, Sun XF, Deavila J, Du M, Zhu MJ. Grape pomace inhibits colon carcinogenesis by suppressing cell proliferation and inducing epigenetic modifications. J Nutr Biochem. 2020;84:108443. doi: 10.1016/j.jnutbio.2020.108443.

2. Yan X, Huang Y, Zhao JX, Rogers CJ, Zhu MJ, Ford SP, et al. Maternal obesity downregulates microRNA let-7g expression, a possible mechanism for enhanced adipogenesis during ovine fetal skeletal muscle development. Int J Obes. 2013;37(4):568-75. doi: 10.1038/ijo.2012.69.
