# Supplementary figures and images for: AMPK Deficiency Increases DNA Methylation and Aggravates Colorectal Tumorigenesis in AOM/DSS Mice
Source: Genes (Basel). 2024 Jun 25;15(7):835. doi: 10.3390/genes15070835 (PMC11276171; doi:10.3390/genes15070835)

## Slide 1
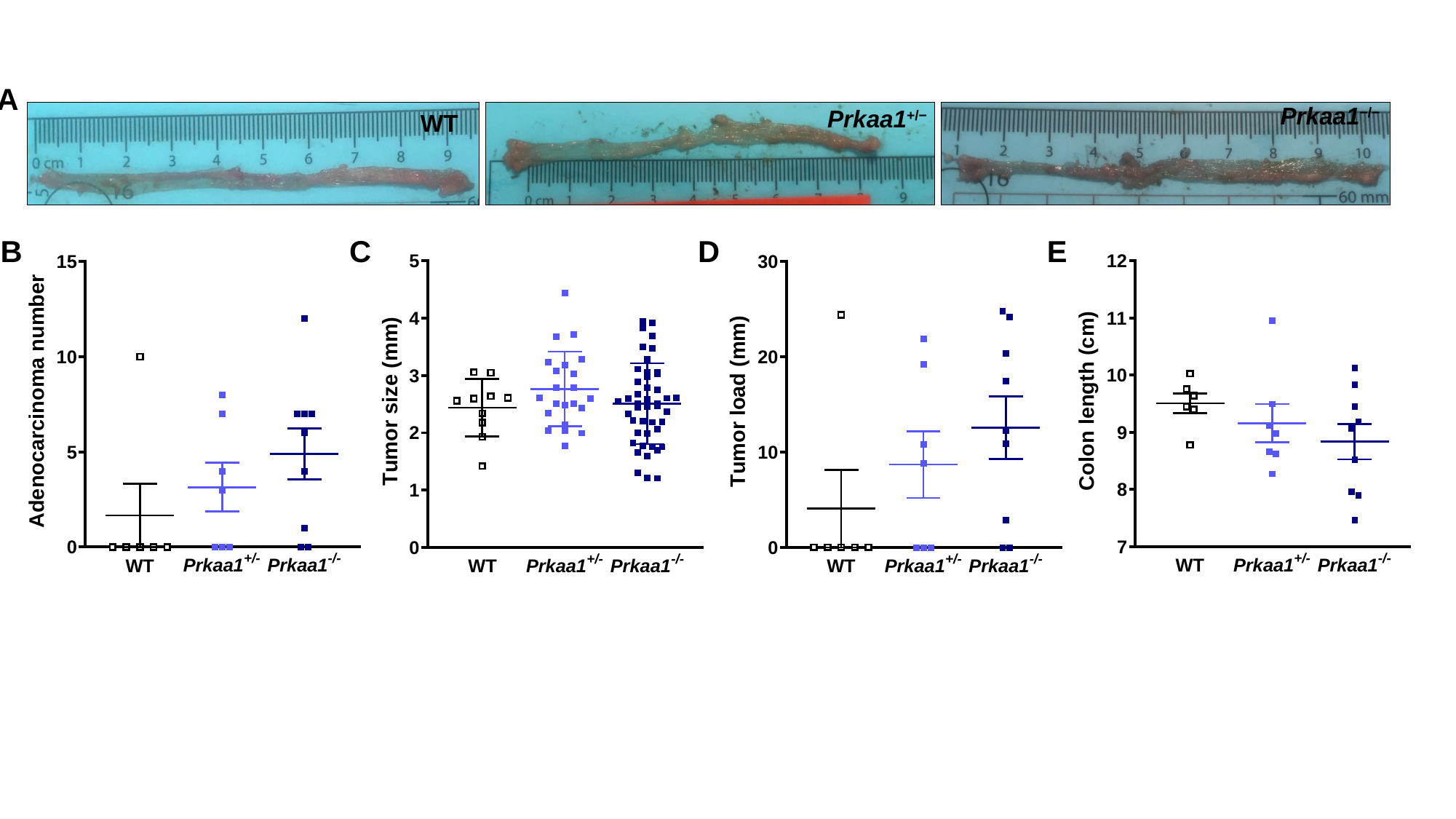

A
Prkaa1−/−
Prkaa1+/−
WT
B
C
D
E

Supplement: Supplementary file 1 [file genes-15-00835-s001.zip › Figure S1.pptx]
